# Atrophy of skin-draining lymph nodes predisposes for impaired immune responses to secondary infection in mice with chronic intestinal nematode infection

**DOI:** 10.1371/journal.ppat.1007008

**Published:** 2018-05-17

**Authors:** Xiaogang Feng, Cajsa Classon, Graciela Terán, Yunlong Yang, Lei Li, Sherwin Chan, Ulf Ribacke, Antonio Gigliotti Rothfuchs, Jonathan M. Coquet, Susanne Nylén

**Affiliations:** 1 Department of Microbiology, Tumor and Cell Biology (MTC), Karolinska Institutet, Stockholm, Sweden; 2 Department of Cellular and Genetic Medicine, School of Basic Medical Sciences, Fudan University, Shanghai, China; 3 Department of Physiology and Pharmacology, Karolinska Institutet, Stockholm, Sweden; Uniformed Services University of the Health Sciences, UNITED STATES

## Abstract

Intestinal nematodes suppress immune responses in the context of allergy, gut inflammation, secondary infection and vaccination. Several mechanisms have been proposed for this suppression including alterations in Th2 cell differentiation and increased Treg cell suppressive function. In this study, we show that chronic nematode infection leads to reduced peripheral responses to vaccination because of a generalized reduction in the available responsive lymphocyte pool. We found that superficial skin-draining lymph nodes (LNs) in mice that are chronically infected with the intestinal nematode *Heligmosomides polygyrus*, do not reach the same cellularity as worm-free mice upon subsequent BCG infection in the skin. B cells and T cells, all declined in skin-draining LN of *H*. *polygyrus*-infected mice, resulting in LNs atrophy and altered lymphocyte composition. Importantly, anti-helminthic treatment improved lymphocyte numbers in skin-draining LN, indicating that time after de-worming is critical to regain full-scale LN cellularity. De-worming, and time for the skin LN to recover cellularity, also mended responses to *Bacille Calmette-Guerin* (BCG) in the LN draining the footpad injection site. Thus, our findings show that chronic nematode infection leads to a paucity of lymphocytes in peripheral lymph nodes, which acts to reduce the efficacy of immune responses at these sites.

## Author summary

Infections with intestinal nematodes may be one explanation to why BCG vaccination is less effective in areas of high worm burden. In support of this, we recently showed that chronic intestinal nematode infection resulted in reduced immune responses and higher mycobacterial burden at distal sites. How a gut-dwelling nematode modulate immune responses in skin-draining lymph nodes (LN) was not clear. We found a reduced expansion of LN draining the BCG injected footpad in worm-infected animals, but no evidence for a spread of regulatory cells or cytokines to the BCG-draining LN. Interestingly, we found that mice chronically infected with intestinal worms had significantly smaller skin-draining LN. We propose that the expansion of mesenteric lymph nodes (mLN) occur at the cost of other LN, leading to atrophy of skin-draining LN. Expansion of the lymphocyte pool by IL-7, allowed worm-infected animals to maintain larger skin-draining LN while the mLN did not further expand. De-worming treatment of mice eventually restored the cellularity of skin-draining LN. This, however, took time indicating that effect of worms persisted long after the infection cleared. By de-worming and allowing time for the LN to recover, the cellular responses to BCG injection in the footpad were restored in the draining popliteal LN. Thus, paucity of lymphocytes at peripheral sites can explain impaired peripheral immune responses in worm-infected animals.

## Introduction

Infections with intestinal nematodes often become chronic in mammals due to both the longevity of worms and continuous reinfection. Worm infections, once chronically established, typically cause little overt pathology but may have implications on growth development, nutritional status and the ability to mount secondary immune responses. Suppressed lymphocyte responses in individuals with worm infections were described around 40 years ago and have since been confirmed in both infectious and autoimmune disease animal models [1–4]. Importantly, de-worming people prior to vaccination may be a strategy to improve vaccine and therapeutic efficacy in populations with high worm-burden [5–7].

Worms have been well-characterised to induce potent Th2 cell responses, typically characterised by the secretion of cytokines such as IL-4 and IL-13, which promote nematode clearance in a number of ways [8]. Viruses and bacterial species including mycobacteria induce Th1 cell responses, which help to eliminate these intracellular pathogens. These two arms of the CD4 T cell response are antagonistic to one another, and as such, one presumption has been that Th2-biased chronic worm infections prevent the efficient development of robust Th1 immunity, required for example, in mycobacteria vaccine responses [9]. Furthermore, helminths are also potent inducers of regulatory T cell (Treg) responses, which have a generalized inhibitory effect on immune function [10]. Downregulation of inflammatory immune responses is essential to minimize pathology in the host, but benefits the survival and chronic establishment of the worm. Despite the regulatory responses evoked by intestinal worms, inflammatory reactions are not completely abolished and a persistent worm infection will provide a continuous supply of antigens that maintain stimulation to the immune system [11].

Many of the stereotypic immune characteristics of a helminth infection including Th2 cells, hallmarked by their production of IL-4, IL-5 and IL-13, regulatory Foxp3+ T cells producing TGF-β and IL-10, goblet hyperplasia, mucus production, mast cell activation and differentiation of M2 macrophages [12] occur in proximity to the worm infection. Thus, the immune responses that directly could counteract responses to vaccination and / or co-infections are primarily compartmentalized to the gut and associated lymphoid tissue. However, human studies as well as our own experimental data show that intestinal nematodes have inhibitory effects on immune responses to BCG immunization given in the skin [2,7]. Thus, it is evident that an intestinal worm also has widespread systemic effects. It is not clear how these distal inhibitory effects are mediated.

Herein we comprehensively analysed how distal skin-draining LN are affected by chronic intestinal worm infection. We found no evidence that Th2 or regulatory T cells disseminated throughout the organism, such that they would directly inhibit immune responses to BCG infection in the skin. Instead, our data support the notion that intestinal worms cause atrophy of skin-draining LN. Skin-draining LN, as they decrease in cellularity, also change in composition having a lower T cell/B cell ratio. Upon secondary immunization or infection, the LN draining the second inoculation site is smaller in worm-infected compared to worm-free mice. These LN, being smaller, have a reduced capacity to recruit and retain cells, resulting in a diminished expansion in response to secondary immunization/infection. Administration of recombinant IL-7 (rIL-7), which increases the survival of lymphocytes and supports an expansion of the total lymphocyte pool [13], allowed worm-infected mice to maintain a larger cell number in skin-draining LN without additional expansion of mesenteric LN (mLN). De-worming of mice led to a recovery of distal LN cellularity, as the mLN contracted. Our findings suggest that the distal immune suppressive effects of intestinal nematodes are not due to specific effects of regulatory and Th2 responses evoked by worms, but rather a broader reconfiguration of the lymphoid system. Our data also explain why BCG vaccination may be less effective in individuals with intestinal helminths and suggest that de-worming may recover cellular responses. However, some time may be needed before effects of de-worming can be beneficial.

## Results

### Cellularity of BCG reactive skin-draining LN is compromised in mice with chronic *H*. *polygyrus* infection

**Fig 1:**
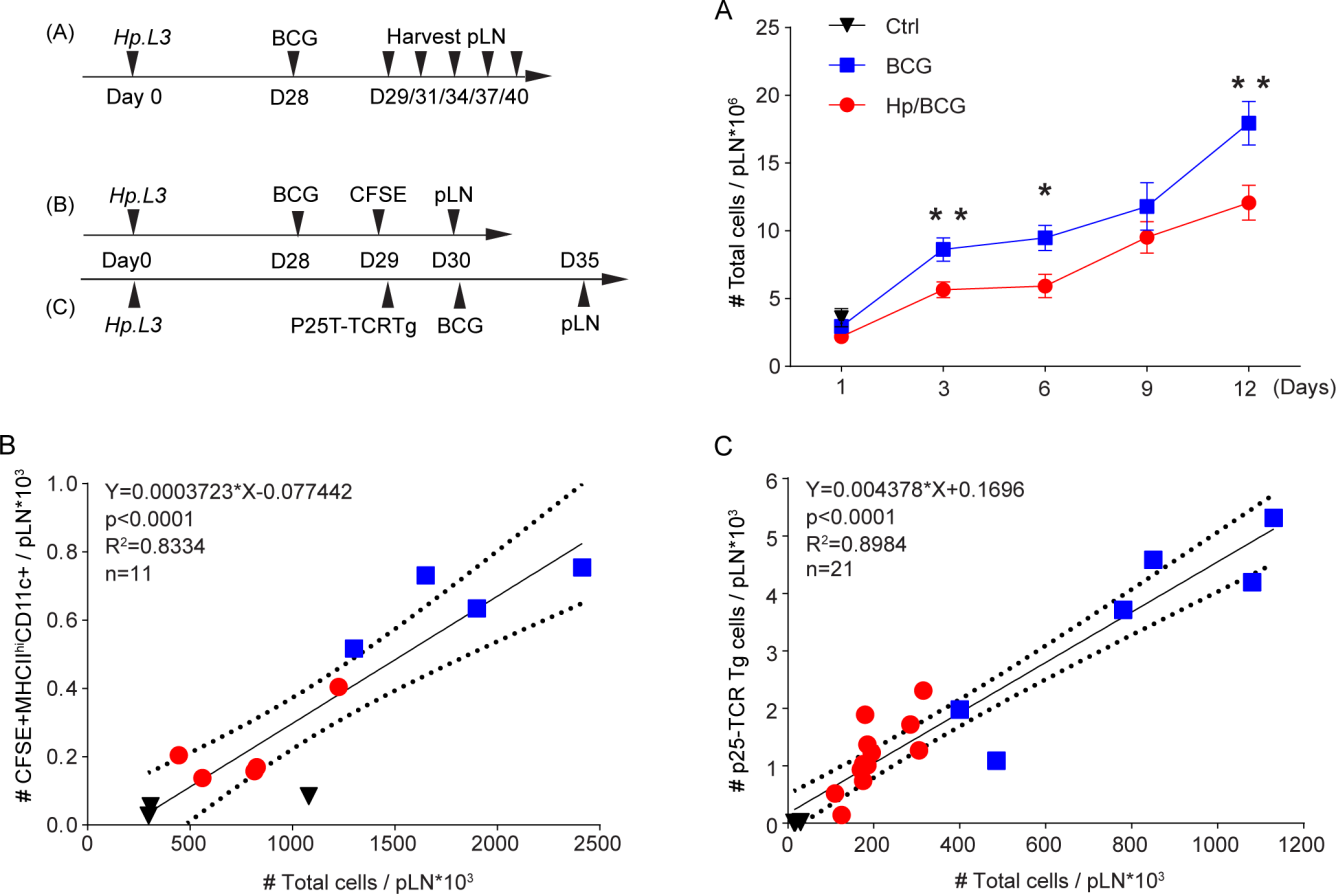
Impaired expansion of popliteal lymph node post BCG injection in mice with *H*. *polygyrus*. C57BL/6 mice (**A, B**) or Ly 5.1 mice (**C**) with chronic *H*. *polygyrus* were injected with 1x10^6^ CFU of *Bacille Calmette-Guérin* (BCG) in the skin of the footpad. (**A**) The expansion of the foot draining popliteal LN was evaluated at different time points. **(B)** Correlation between the cellularity of the popliteal LN and the number of migratory (MHC-II^hi^ CD11c+) dendritic cells tracked by CFSE between 48 and 72 hours after BCG injection **(C)**. Correlation between the number of mycobacteria specific congenic P25-TCRTg-ECFP T cells 6 days after BCG infection and the cellularity of the popliteal LN. In B and C, the numbers of cells were determined by FACS. Data in B is representative of two or more experiments and in C pooled from two replicate experiments. 1x10^5^ P25-TCRTg-ECFP cells were injected *i*.*v*. the day before BCG infection. Parts of the data used to generate Fig 1B and 1C have also been use in a previous publication [2].

We have previously reported that mice with chronic *H*. *polygyrus* infection have reduced cellular responses when subsequently infected with BCG [2]. To see how the BCG response developed in worm-infected and worm-free mice respectively, we examined the cellular expansion of the reactive popliteal LN (pLN) following infection with BCG in the footpad. In line with our previous observations, LN expansion in response to BCG was reduced in mice with chronic *H*. *polygyrus* co-infection and did not reach the cellularity of worm-free mice during 12 days (Fig 1A).

In response to BCG footpad infection, fewer dendritic cells (DCs) migrated from the footpad to the draining popliteal LN (pLN) in worm-infected mice compared to worm-free mice. Likewise, fewer mycobacteria specific CD4 T cells (P25-TCRTg cells) were found in the pLN of worm-infected compared to worm-free mice after BCG infection.

Although, there was a difference in the total number cells in response to BCG infection neither the migratory DCs nor the mycobacteria-specific P25-TCRTg cells appear to be specifically targeted. The frequencies of afore mentioned cells were in fact similar in pLN of worm-infected and worm-free mice infected with BCG in the footpad. When further calculated, it was evident that both the number of DCs migrating from the skin to the draining LN 48–72 hours after BCG infection (Fig 1B) and the number of P25-TCRTg T cells measured 6 days after infection (Fig 1C) are directly proportion to the total cellularity of the lymph node. This correlation was independent of worm infection.

### Th2 and Treg cells are not enriched in skin-draining LN

**Fig 2:**
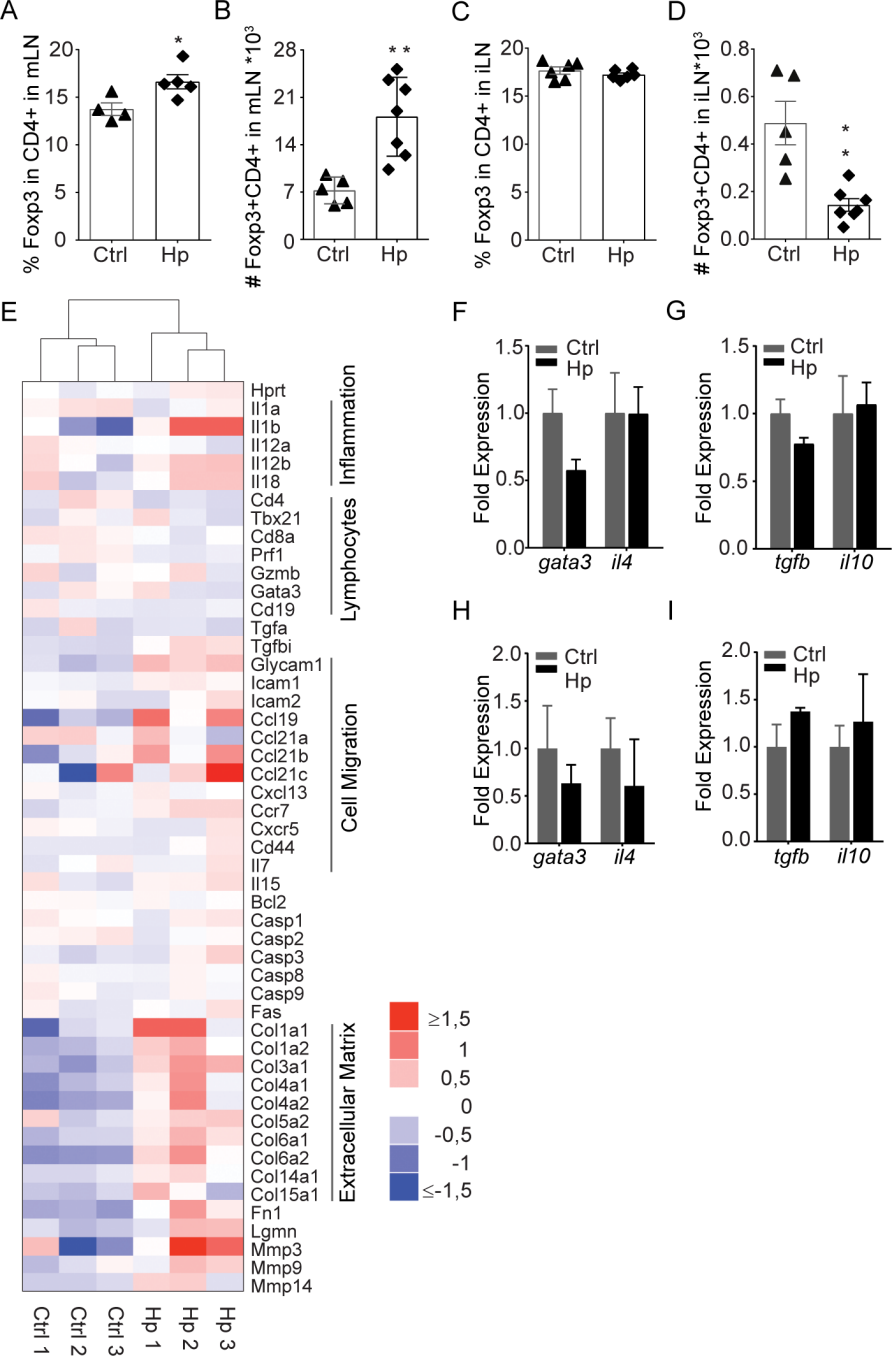
Regulatory immune responses are compartmentalized. Foxp3+CD4+ cells in LN from C57BL/6 mice with chronic *H*. *polygyrus* infection. Percentage of Foxp3+ **(A, C)** and number of CD4+Foxp3+ cells **(B, D)** in the mLN **(A, B)** and iLN **(C, D).** Non-clustered heatmap of RNA sequencing data (Log_2_ FPKM values normalized to the row median) from individual animals for genes with assumed association to inflammation, lymphocytes, cell migration, cell death/survival and extra cellular matrixed **(E)**. Relative gene expression of Th2 **(F, H)** and regulatory proteins **(G, I)** in iLN **(F, G)** and spleen **(H, I)** as determined by qPCR. The fold change as expressed by 2^-ΔΔCT^ is shown. Data is representative of two or more experiments.

The induction of regulatory T cells (Treg) has been proposed as a major mechanism by which worms down-modulate host immunity [4]. To assess whether *H*. *polygyrus* induced widespread systemic immune suppression, we evaluated Treg and cytokine expression in skin-draining LN. As expected, an increase in regulatory Foxp3^+^ CD4^+^ T cells was evident in the mLN following *H*. *polygyrus* infection (Fig 2A and 2B). This increase did not extend to skin-draining LN where the frequency of Foxp3-expressing CD4+ cells was unchanged and cell numbers actually decreased (Fig 2C and 2D). In line with this, RNA sequencing of distal LN in control or chronic *H*. *polygyrus* infection did not reveal any specific up- or down-regulation of Th2- or Treg-associated genes (Fig 2E). Further, targeted RT-PCR analysis of relative gene expression associated with worm-induced immune responses did not show up-regulation of gene expression associated with Th2 (GATA-3, IL-4) or Treg cells (IL-10, TGF-β) in skin-draining LN of *H*. *polygyrus-*infected mice (Fig 2F and 2G).

Others have indicated that regulatory and Th2 responses induced by the worm are evident in the spleen [14]. We do not exclude that this may occur, but our data does not support heightened Treg or Th2 responses in the spleen. We have shown previously [2] that frequencies and numbers of CD4+Foxp3+ T cells are similar in the spleens of worm-infected and worm-free mice. In line with this, we here show that splenic expressions of selected Th2 and Treg genes were similar *H*. *polygyrus* infected and control mice (Fig 2H and 2I).

### *H*. *polygyrus* infection causes a progressive atrophy of skin-draining LN that stabilises with time

**Fig 3:**
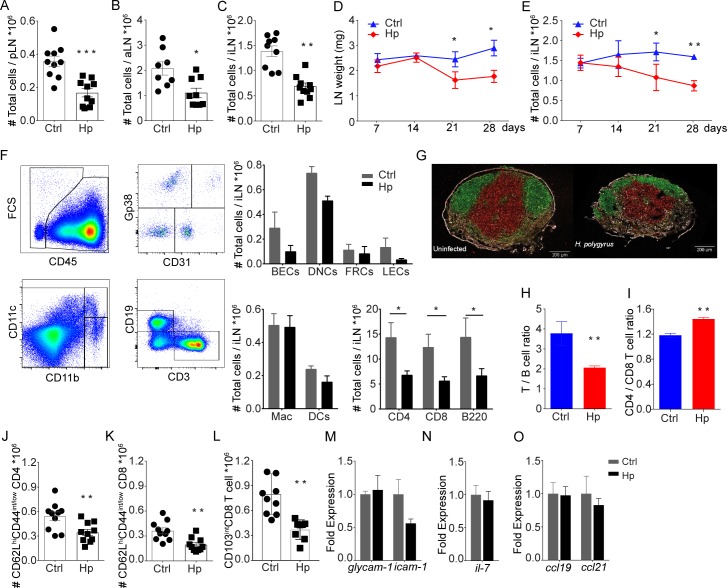
Cellularity and lymphocytes composition in non-draining skin LN distal to *H*. *polygyrus* infection. Cellularity of superficial popliteal **(A),** axillary **(B)** and inguinal **(C)** LN in mice chronically infected with *H*. *polygyrus*. Results show data from two pooled experiments. Development of iLN weight **(D)** and cellularity **(E)** following infection of C57BL/6 mice with *H*. *polygyrus*. Composition of iLN cells as determined by FACS analysis of lymphocyte and stromal cell subsets in mice with and without chronic (28 days) *H*. *polygyrus* infection **(F)**. Confocal image of popliteal LN from naïve (left) and *H*. *polygyrus* infected (right) mice stained for T cells/CD3 (green), B cells/B220 (red) and Collagen 4 (white) **(G)**. Ratio of T / B lymphocytes **(H)** and CD4 / CD8 T cell **(I)** in uninfected and mice with chronic *H*. *polygyrus* infection. Numbers of naïve (CD62L^hi^CD44^int/low^) CD4+ **(J)** and CD8+ T cells **(K)** and CD103^int^ CD8 T cells **(L)**. Cell were gated on CD3+ singlets as shown in S4 Fig. Relative expression of GlyCAM-1 and ICAM-1 **(M)**, IL-7 **(N)**, as well as CCL19 and CCL21 **(O)** in iLN. Data is representative of two or more experiments.

We then sought to understand if other underlying changes in the superficial LN could explain the diminished responses to secondary infection/vaccination in the skin. The RNA-seq analysis pointed to a reduced lymphocyte cellularity and an increased expression of genes associated with matrix regulation in inguinal LN (iLN) of worm-infected mice (Fig 2E). Consistent with this, we found the cellularity of skin-draining LN was less in mice with chronic *H*. *polygyrus* infection (Fig 3A–3C). Both weight and cellularity were gradually reduced in iLN from *H*. *polygyrus-*infected mice (Fig 3D and 3E), becoming significant three weeks after infection, partially in line with previous work [15]. At the time when the *H*. *polygyrus* infection was considered chronic (28 days post infection) [12,16], LN cellularity did not change further. That said, the atrophy of skin-draining LN was then preserved and still evident three months after *H*. *polygyrus* infection (S1A Fig). The effect of *H*. *polygyrus* infection on skin-draining LN was partially dose-dependent, a lesser effect was observed following infection with 50 *L3* while giving 100 *L3* almost had the same effect as 200 *L3* (S1B Fig). Smaller skin-draining LN observed in *H*. *polygyrus*-infected mice could not be explained by impaired growth development since infected mice gained weight normally (S1C Fig) and consumed similar amounts of food as non-infected mice (S1D Fig). Further, skin LN atrophy was not a consequence of altered immune cell distribution in growing mice since smaller skin-draining LN were also evident in adult (9 weeks old at time of infection) mice infected with *H*. *polygyrus* (S1E Fig).

The overall loss of cellularity in skin-draining LN of *H*. *polygyrus*-infected mice was primarily in lymphocytes (Fig 3F). Stromal and myeloid cells were not significantly affected, though the trend was toward a decrease also in these populations (Fig 3F). Histopathological evaluation of superficial LN did not point to any major structural changes in *H*. *polygyrus* infected mice (Fig 3G). On the other hand, the effect on lymphocyte subsets was not equally distributed. More T cells than B cells were lost and viewed as frequencies CD8+ T cells were lost over CD4+ T cells, causing an alteration in T cell/B cell as well as CD4+/CD8+ T cell ratio in skin-draining LN of *H*. *polygyrus*-infected compared to worm-free mice (Fig 3H and 3I). Analysis of CD44, CD62L and CD103 on T cells from skin-draining LN show that *H*. *polygyrus* infection caused a general reduction in T cells, although the loss of naïve T cells (CD4+CD62L^hi^ CD44^int/low^ and CD8+ CD62L^hi^ CD44^int/low^ as well as CD8+ CD103^int^ T cells) was most evident (Fig 3J–3L, S1F–S1O Fig). This suggests that naïve lymphocytes primarily are lost from skin-draining lymph nodes in chronic worm infection.

Considering that naïve lymphocytes were more affected than other subsets, we measured the expression the integrin GlyCAM-1 and ICAM-1 as well as the chemokines CCL19 and CCL21, and the cytokine IL-7, since all are important in recruitment and maintenance of lymphocytes in the skin-draining LN [17]. However, we found no change in the relative expression of these molecules in iLN between *H*. *polygyrus*-infected and uninfected mice (Fig 3M–3O).

### Fluctuating blood lymphocyte levels following *H*. *polygyrus* infection

**Fig 4:**
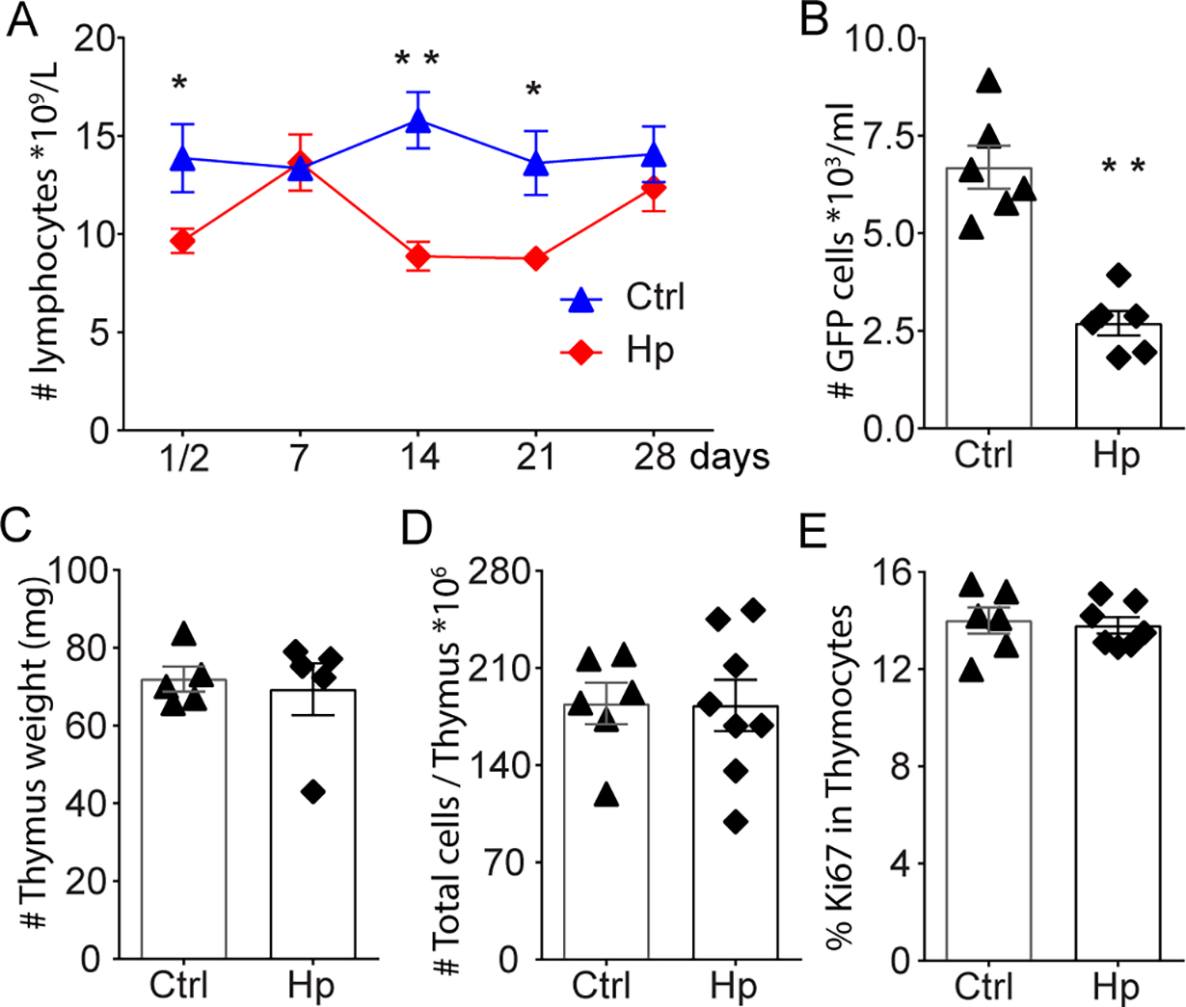
Fluctuation in circulating lymphocytes following *H*. *polygyrus* infection. Lymphocyte concentration in peripheral blood following *H*. *polygyrus* infection as determined by an automated haematology analyser **(A)**. Detection of transferred (GFP+) lymphocytes in blood as determined by FACS **(B)**. Thymic weight **(C)** and cellularity **(D)** and frequency of proliferating, Ki67+ **(E)** in mice with or without chronic *H*. *polygyrus* infection. Data is representative of two or more experiments.

The maintenance of LN cellularity is mainly determined by input and output of circulating lymphocytes between lymphoid organs and blood [18]. Thus, smaller skin-draining LNs may reflect changes in the concentration of circulating blood lymphocytes. To address this we followed blood lymphocyte counts following *H*. *polygyrus* infection. In support that the smaller LN are a consequence of decreased lymphocyte trafficking, we found that the levels of circulating lymphocytes drop in mice following *H*. *polygyrus* infection (Fig 4A). While the decline of cellularity in skin-draining LN appeared continuous, assessment of blood lymphocytes indicated that the reduction might occur in waves. Blood lymphopenia during *H*. *polygyrus* infection was most evident in the first days after infection and 2–3 weeks after infection with a period of normal values in between (Fig 4A). The drops in blood lymphocyte levels coincided relatively well with described waves of innate and Th2 immune responses towards the worm in C57BL/6 mice and accompanying enlargement of the reactive mLN [16]. Upon transfer of lymphocytes from an uninfected mouse, fewer donor cells were found in the circulation of the worm-infected compared to the worm-free recipient mice 4 hours after transfer (Fig 4B). This indicated that lymphocytes might be more rapidly removed from the circulation in mice with chronic *H*. *polygyrus* infection.

The superficial LN atrophy and lymphopenia did not appear to be coupled to thymic involution observed in other infections [19–22], since the weight and cellularity of the thymus as well as Ki67 staining of thymocytes were not changed by chronic *H*. *polygyrus* infection (Fig 4C–4E).

### Expansion of the mesenteric LN and retention of lymphocytes

**Fig 5:**
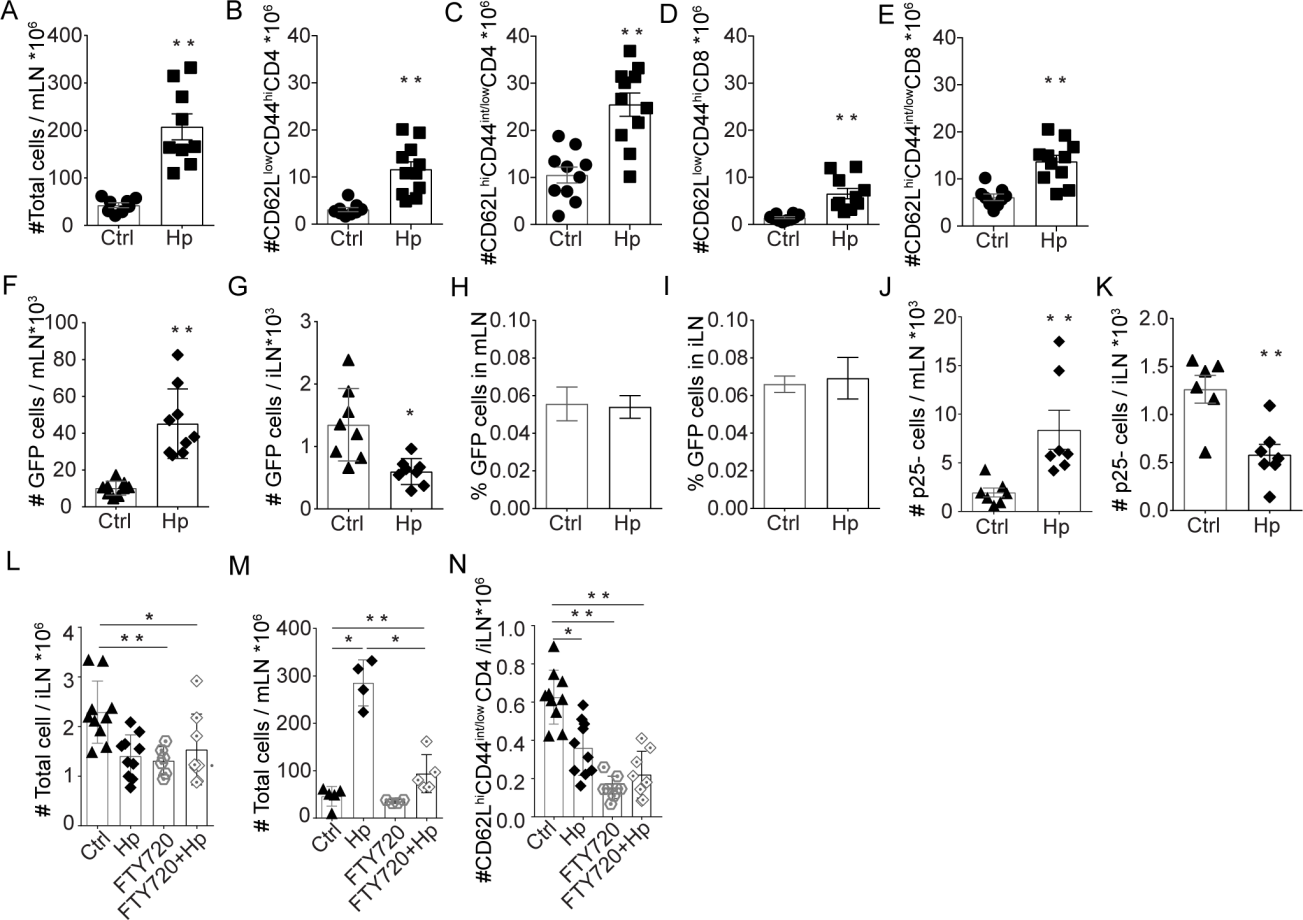
Accumulation of cells in mLN after *H*. *polygyrus* infection. Lymphocyte numbers in mLN of C57BL/6 mice with or without chronic *H*. *polygyrus* infection **(A)**. Analysis of CD4 and CD8 T cells based on the expression of CD62L^low^CD44^hi^ **(B, D)** and CD62L^hi^CD44^int/low^ **(C, E)** using FACS. Numbers **(F, G)** and frequency **(H, I)** of GFP+ lymphocyte 4 hours after transfer as determined by FACS analysis of recipient LN suspensions. Tracking of “naïve” P25-TCRTg (CD45.2) T cells in recipient LN: P25-TCRTg cells were transferred to congenic Ly 5.1 (CD45.1) prior to *H*. *polygyrus* infection. Lymph nodes were collected 21 days after *H*. *polygyrus* infection and the number of P25-TCRTg cells in mLN **(J)** and iLN **(K)** were determined by FACS. In F-I, mice received 1x10^6^ cells isolated from peripheral LN of naïve C57BL/6-GFP mice and in J and K recipient mice received 1x10^6^ LN cells isolated from P25-TCRTg EGFP mice. Influence of circulating cells on maintenance of LN cellularity: Mice treated with FTY720 throughout the course of *H*. *polygyrus* infection were sacrificed 4 weeks after infection and the cellularity of iLN **(L)** and mLN **(M)** were determined. Naive CD4+ T cell in iLN from mice with or without FTY720 treatment (**N**). Data is representative of two or more experiments.

The mLN draining the intestine displayed a 5-fold increase in cellularity in mice chronically infected with *H*. *polygyrus* compared to uninfected mice (Fig 5A). The cellularity of the spleen was on the other hand not significantly changed (S2A Fig). While the majority of CD4+ T cells in mLN of infected mice had a naïve phenotype (CD62L^hi^CD44^int/low^), as expected, more effector (CD62L^low^CD44^hi^) cells were also found in worm-infected animals compared to worm-free mice (Fig 5B–5E). To test if naïve cells were “trapped” in the mLN of infected mice, we transferred total labelled lymphocytes from uninfected animals into recipients with or without chronic *H*. *polygyrus* infection. We tracked these transferred cells and found a higher number in mLN and a lower number in skin-draining LN of *H*. *polygyrus*-infected compared to control mice 4 hours after transfer (Fig 5F and 5G). However, when viewed as frequency, it was evident that the relative numbers and the cell subsets entering the superficial LN were similar and proportional to the total cellularity of the various LN (Fig 5H and 5I, S2B–S2F Fig). Similar to that observed in skin-draining LN, the frequency of transferred T cells found in mLN were not altered by the worm infection, and only reflected the proportion of these subsets in the transferred population (S2G, S2H, S2J and S2K Fig), while the frequency of transferred B cells were slightly higher in mLN (S2I Fig). Tracking of labelled cells 12 hours after transfer showed a similar distribution in skin-draining LN and mLN as during 4 hours (S2L–S2O Fig).

To test how cells with antigen specificity unrelated to the worm would distribute in *H*. *polygyrus*-infected animals over time, we transferred mycobacteria Ag85B-specific P25-TCRTg cells to mice prior to worm infection. As was the case for the transfer of total lymphocytes (Fig 5F and 5G), P25-TCRTg cells were maintained in higher numbers in mLN and in lower numbers in skin-draining LN of mice with chronic worm-infection compared to uninfected mice (Fig 5J and 5K).

Our data show that skin-draining LN become atrophic as the worm infection progresses and the mLN expand. This could be an effect of fewer circulating cells reaching the skin-draining LN, possibly being sequestered to the enlarged mLN and the intestine. In an attempt to assess the impact of circulating lymphocytes on LN cellularity during the span of experimental *H*. *polygyrus* infection, we systemically blocked lymphocyte egress throughout the course of the infection using the S1P1 agonist FTY720. This treatment removes lymphocytes from the circulation and prevent cells from leaving the bone marrow, the thymus and LN [23,24]. Long-term treatment with FTY720 is quite safe, the toxic effects, apart from blocking lymphocyte egress are mainly on cancerous cells leaving normal cells intact. As expected, much fewer lymphocytes were detectable in blood after FTY720 treatment (5–6 times less compared to untreated). The FTY720 treatment alone, given over 4 weeks, caused a generalized reduction in cellularity in both mLN and iLN (Fig 5L and 5M). After 4 weeks of FTY720 treatment the skin-LN cellularity was about 50% of that in untreated mice regardless of *H*. *polygyrus* infection or not (Fig 5L). The mLN in *H*. *polygyrus-*infected and FTY720-treated mice were expanded (Fig 5M), albeit much less compared to the untreated *H*. *polygyrus* infected mice (Fig 5M). As anticipated FTY720 dramatically affected naïve cells in particular CD4 T cells (Fig 5N). Weight gain and worm-load was similar in FTY720 treated and untreated animals. We believe these results reflect the requirement of a constant supply of circulating naïve lymphocytes for maintenance and expansion of LN, in presence or absence of worm infection.

### Recovery of skin LN cellularity and responses to BCG

**Fig 6:**
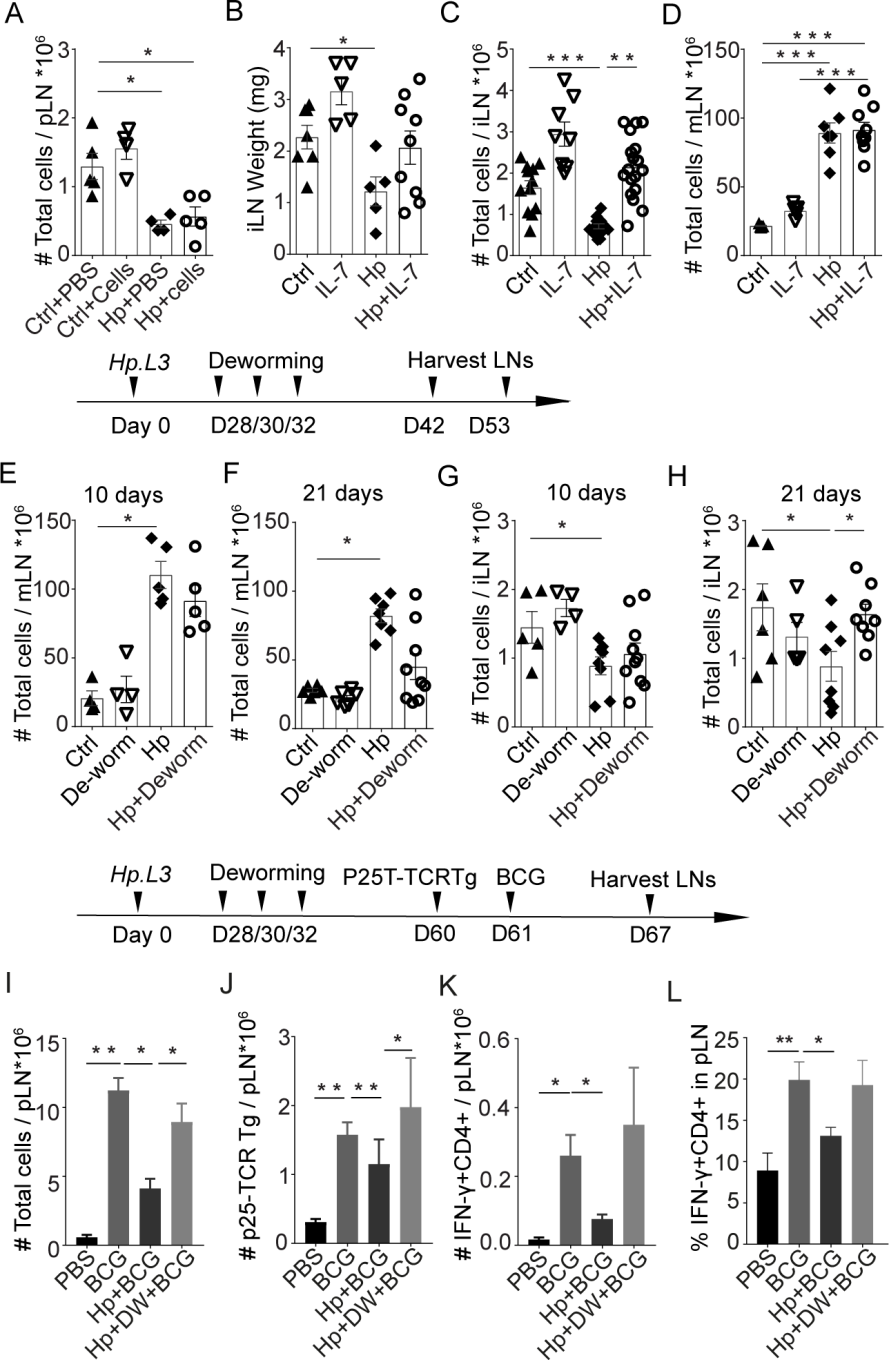
Recovery of iLN cellularity and ability to mount CD4 T cell responses to BCG. Addition of cells by transfer of lymphocytes cannot rescue iLN atrophy in *H*. *polygyrus* infected mice: Single cells LN suspension (7-10x10^6^ cells) from uninfected C57BL/6 mice were injected into *H*. *polygyrus* infected or worm free recipient mice every third day for three weeks starting the day after infection. The cellularity of popliteal LN (pLN) was determined four weeks after *H*. *polygyrus* infection **(A).** Superficial LN cellularity can be maintained by IL-7 treatment: Mice infected with *H*. *polygyrus* were treated with rIL-7 every other day for total four weeks. Weight of iLN **(B)** and cellularity of iLN **(C)** and mLN **(D)** was determined at the end of therapy. Cellularity of iLN is recovered following de-worming: C57BL/6 mice with chronic *H*. *polygyrus* were dewormed or left untreated. The reversion of changes in mLN **(E, F)** and iLN **(G, H)** cellularity was determined 10- days **(E, G)** and 21- days **(F, H)** after deworming. T cells responses to BCG infection can be corrected by deworming and recovery of cellularity: C57BL/6 mice with chronic *H*. *polygyrus* infection (Hp) were dewormed (DW) or not. Control mice (BCG and PBS) received anti-helminthic therapy at the same time. Four weeks later, mice were injected with 1x10^6^ CFU BCG in the footpad or mock treated with PBS. 1x10^6^ P25- TCRTg EGFP cells were transferred *i*.*v*. to recipient mice the day before BCG injection. Popliteal LN were harvested 6 days after BCG injection and the total number of LN cells (**I**), P25-TCRTg cells tracked by EGFP (**J**) and IFNγ producing CD4+ T cells following PMA/Ionomycin restimulation **(K, L)** were determined. Data is representative of two or more experiments.

To test if the loss of cellularity in superficial LN could be compensated for in *H*. *polygyrus*-infected mice, we transferred large numbers of lymphocytes at regular intervals following infection. This procedure did not alter cellularity in superficial LN from worm-infected or uninfected animals (Fig 6A). This suggests that in the case of chronic infection, lymphoid homeostasis is altered to accommodate for the expansion of the mLN, at the expense of the cellularity of peripheral LN, and that this peripheral deficit cannot be rescued simply by providing more cells. We then questioned whether we could restore peripheral LN cellularity by supplying factors involved in lymphoid homeostasis. To this end, we administered recombinant IL-7 to mice throughout the course of *H*. *polygyrus* infection. The idea behind the IL-7 supplementation was to allow a larger lymphocyte pool. IL-7 is essential for survival of lymphocytes and limitations in IL-7 could prevent the lymphocyte pool from expanding and be a regulating factor in maintenance of LN cellularity [13]. rIL-7 treatment led to an increase in cellularity and recovery of T and B the iLN (Fig 6B and 6C, S3A–S3H Fig) while the mLN did not expand further in *H*. *polygyrus*-infected mice (Fig 6D), supporting the notion that the size of the lymphocyte pool may be a limiting factor in determining LN size both during steady state and infection.

Killing of parasitic worms have been proposed as a strategy to improve vaccine efficacy [5,25]. To test if removal of worms would lead to recovery of LN cellularity we treated mice chronically infected with *H*. *polygyrus* with three doses of pyrantel pamoate over 5 days. LN cellularity was assessed 10- and 21- days after therapy. De-worming removed worms within 2–3 days, as determined by the lack of eggs in faecal samples. The enlargement of the mLN persisted for weeks (Fig 6E and 6F). This we propose can have implications on skin-draining LN, which remained smaller, compared to control mice and similar in cellularity and composition to mice infected with worms 10 days after de-worming (Fig 6G and S3I–S3P Fig). Three weeks after anti-helminthic treatment, the cellularity of iLN had increased and the number of T and B cells were similar to that of control mice (Fig 6H, S3Q–S3X Fig). We then addressed if de-worming and time given for the LN to regain cellularity would correct the impaired responses to BCG previously shown in Fig 1 and [2]. Indeed, we found that responses to BCG were returning and were similar in de-wormed and worm free animals (Fig 6I–6L). Indicating that de-worming, given time, positively affect the ability to mount antigen specific response to immunization in the skin.

## Discussion

Chronic helminth infections are implicated in impaired responses to vaccination and control of secondary infections such as tuberculosis [9,26,27]. We have previously shown that mice with chronic *H*. *polygyrus* infection have muted responses to BCG and *Leishmania* infection [2]. Th2 and regulatory T-cell responses are typically depicted as the culprits causing the worm-mediated suppression of Th1-controlled infections [27–31]. However, we find no evidence for disseminated up-regulation of Th2 or regulatory cytokines in skin LN. Nor is there a widespread increase in regulatory T -cells in our model [2] (Fig 2F and 2G). Instead, our data suggests that distal immune suppression in worm-infected individuals may be a consequence of redistribution and competition for the available lymphocyte pool. Our interpretation is supported by a recent study showing that naïve lymphocytes accumulate in the mLN and are lost from peripheral LN in worm-infected mice [15]. Similar to our observations using BCG, these mice displayed muted responses and impaired control of influenza infection.

Impaired immune responses to skin-BCG infection can thus be an effect of reduced cellularity of the BCG draining node. There is a positive correlation between LN size and response to BCG independent of worm infection. Mice with intestinal worms, which have smaller skin-draining LN, accordingly displayed a reduced response to BCG injection in the footpad compared to worm-free mice (Fig 1). The loss of cellularity in skin-draining LN was partially dose-dependent and less obvious when a low-dose worm infection was used (S1B Fig), supporting the notion that negative effects of worms are most evident when the worm-burden is high. Other than size, we found no gross pathological alterations evident macroscopically or by histology in the superficial LN of worm-infected mice. That said, lymphocyte numbers were reduced in the skin-draining LN of worm-infected mice, where furthermore, an increase in B-cell/T-cell and CD4+/CD8+ T-cell ratios were seen. The latter observation likely reflects the turnover of the respective cells subsets within a LN, B cells having a more mature phenotype and thus slower turnover than T cells. Further, naïve cells were lost over memory/effector cells, similar to findings by King *et al*. [15]. How these changes in lymphocytes composition affect subsequent immune responses in detail remain to be determined.

Thymic involution and decreased output of T cells has been reported to occur in infectious as well as inflammatory diseases [21]. The lymphocyte levels fluctuated during *H*. *polygyrus* infection. However, our results suggest that the thymus is normal in mice with chronic *H*. *polygyrus* infection, with similar weight, cellularity and proliferative capacity of thymocytes as that of uninfected animals (Fig 4). Thus, the reduced cellularity in superficial LN is not likely due to reduced thymic output. In addition, we did not find any evidence for increased cell death in skin-draining LN from worm-infected mice and mRNA expressions of caspase-3 were not different between the groups.

The reactive mLN draining the small intestine was greatly enlarged in mice with chronic *H*. *polygyrus* infection (Fig 5A), and remained so for as long as we followed the infection (3 months).

A LN expands in several steps. Upon infection, the draining LN rapidly responds to accommodate an expansion in cellularity. More lymphocytes are allowed to enter the LN through the high endothelial venules and signals that allow lymphocytes to egress from the LN are down regulated resulting in a “LN shut down” [32]. This “trapping” of naïve lymphocytes results in a rapid increase in cellularity of the reactive LN [17,33] serving to increase the probability of naïve T-cell encounter with their cognate antigen on antigen presenting cells thereby facilitating the initiation of an adaptive immune response [33]. The LN then further expand as lymphocytes undergo clonal expansion. In a resolving infection, where the pathogen is cleared, the immune response contracts and the reactive LN returns to normal size. In a chronic intestinal nematode infection this does not occur. Rather, the infection persists and the mLN remain enlarged. The expansion of mLN and the accompanying reduction of skin-draining LN was dependent on the infectious dose (S1B Fig). Thus, the immune response mounted, measured as total cellularity, match the worm load. Interestingly, following de-worming, the mLN remain enlarged for weeks. In viral infections, it has been suggested that antigens can persist for extended time even in the absences of the infection and maintain T cell responses [34]. However, other infections may require persistence of the infection to uphold T cell responses. CD4 T cells appear to require a continuous supply of protein/peptide to remain activated, and following removal of the antigen source the ability of DCs from draining LN to activate antigen specific responses are rapidly lost [35]. While persisting worm antigen and/or reminiscent inflammation may play a part in maintenance of mLN sizes following de-worming, we suggest that the enlarged LN size *per se* is as important. Size dependent distribution and re-distribution of lymphocytes amongst LN, alone, can explain why skin-draining LN loose cellularity and why it takes time to regain peripheral LN cellularity in previously worm-infected mice. Assuming that the total lymphocyte output and pool are relatively constant [36] and, as we show, that lymphocytes distribute amongst LN according to LN cellularity/size our data suggest that the mLN simply by being enlarged will over time cause skin-draining LN to lose cellularity. A larger node can retain more of the circulating lymphocytes. The physical properties that accompanies a larger LN size, such as longer cell retention time in the LN, will be important in maintaining higher cellularity when lymphocyte numbers are limited. Thus, unless more lymphocytes are produced, or survival increased, expansion of one LN will be at the expense of others.

We consider thus, that distribution of circulating lymphocytes is foremost a function of LN size/cellularity. Accordingly, transfer of cells to *H*. *polygyrus* infected mice showed that lymphocytes distribute proportional to the size of skin-draining LN size just as they do in uninfected mice, with fewer transferred cells found in skin-draining LN of worm-infected mice as they are smaller and the enlarged mLN contained more transferred cells compared to uninfected mice (Fig 5F–5K). Thus, the LN size *per se* contribute to regulate the number of cells in a LN during steady state as well as upon chronic infection. Since mLN cellularity decrease slowly following termination of infection, the capacity to retain circulating lymphocytes, while steadily decreasing, can remain for a prolonged period. On this line of thought, resolution of skin LN cellularity after a worm infection is bound to take time, even in the absence of persisting antigens.

Mesenteric LN expansion in response to *H*. *polygyrus* infection is however constrained. We found that, while many more in absolute numbers, the frequencies of transferred cells were actually less in mLN of *H*. *polygyrus* infected mice. This may reflect that counter regulatory mechanisms are in place preventing the mLN to expand overly much. Indeed, we found, in line with previous reports, that the relative expression of both CCL21 and CCL19 mRNAs were down regulated in the reactive mLN and about 50% of that in uninfected mice, [37]. Moreover, treatment with rIL-7, while expanding the superficial LN in worm-infected mice, did not further expand the already enlarged mLN (Fig 6B–6D).

The upkeep of LN is dependent on a continuous supply of new lymphocytes. Without this refill, the number of lymphocytes decline and LN lose size and cellularity, as observed following FTY720 treatment. Limitations in the output of lymphocytes and/or size of the lymphocyte pool may thus explain the skin LN atrophy observed in *H*. *polygyrus* infected mice. However, transfer of large number of lymphocytes, to compensate for the loss of cellularity in superficial LN of *H*. *polygyrus* infected mice, did not alter LN cellularity. This is in line with that lymphocytes, at least under steady state conditions, can only exist in predestined quantities and that the total number of lymphocytes within the circulation and secondary lymphoid tissue is restricted [36].

IL-7 is essential in lymphocyte homeostasis and is a limiting factor in determining the size of the lymphocyte pool [13]. The circulating levels of IL-7 were very low (0–10 pg) and mRNA expression levels in iLN similar in worm-infected and worm-free mice (Fig 3N). Yet, administration of exogenous IL-7 would allow an expansion of the lymphocyte pool and resulted in our system in larger inguinal LN, with restored naïve T cell and B cell numbers, in worm-infected mice (S3A–S3H Fig).

**Fig 7:**
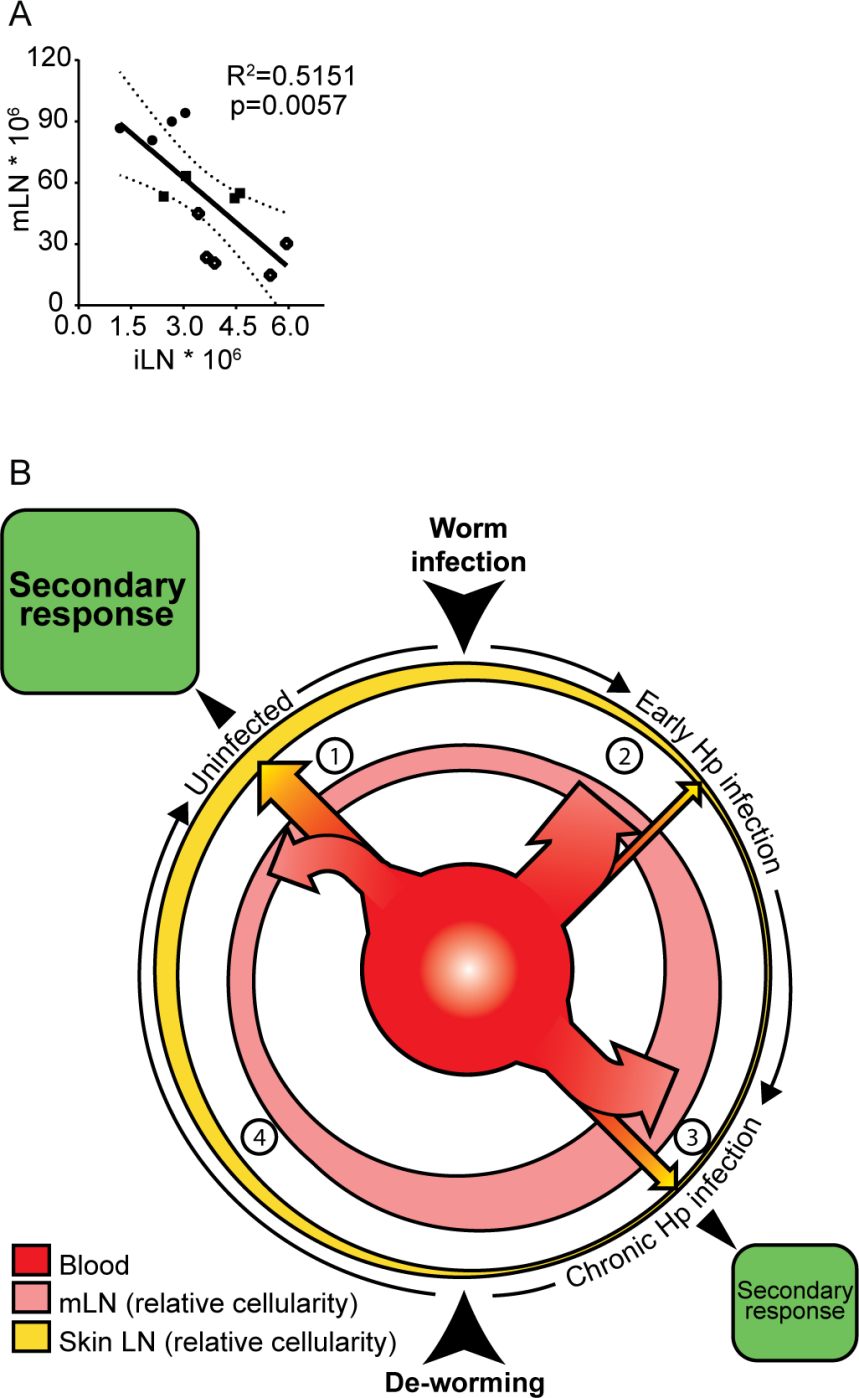
The enlarged mLN in animals with chronic intestinal worms results over time in a loss of lymphocytes from skin-draining LN. There is a negative correlation between the size of iLN and mLN in *H*. *polygyrus* infected mice **(A)** indicating a restriction in the number of lymphocytes available and re-distribution of lymphocytes in animals with chronic worm infection (graph generated from data in S1B Fig). Assuming that the circulating pool of lymphocytes is constant and that the spleen size/cellularity is normal in chronic *H*. *polygyrus* infection, a model of lymphocyte distribution between the reactive mLN and skin-draining LN in animals infected with worms is proposed **(B)**: ***1*.** During “steady state”**,** in a non-infected mouse, circulating lymphocytes distributes amongst LN proportional to the existing LN cellularity *(the outermost yellow circle represents the total skin-draining LN and the pink circle represents mLN*, *the widths of the lines symbolize the relative sizes of LN*). ***2*.** Following *H*. *polygyrus* infection, lymphocytes are recruited from the blood (*red inner circle*) to the reactive mLN, where they are retained. As lymphocytes are activated and go through clonal expansion the mLN expand further. Since the lymphocyte pool is restricted, the increase in mLN size *per se* will facilitate a re-distribution of circulating cells: When a larger proportion of circulating cells are trapped in the mLN (*indicated by a larger arrow going from the blood to the mLN*), the lymphocytes in non-draining skin LN will not be replaced at the same rate as they are lost. Given enough time the skin-draining LN become atrophic. ***3*.** Chronic *H*. *polygyrus* infection maintains enlarged mLN. Since lymphocytes distribute proportional to LN cellularity, more cells distribute to the enlarged mLN and fewer cells enter the smaller skin-draining skin LN (*indicated by the sizes of arrows from the blood to the respective LN*). This new “equilibrium” maintains a lower cellularity in skin-draining LN for as long as the intestinal worm infection persists. Upon a secondary infection in the skin, as an effect of that fewer cells are present in the skin-draining LN, the magnitude of the 2^nd^ immune responses will be reduced in worm infected animals (*indicated by the smaller size of the green box*.) ***4*.** Following de-worming the worms are rapidly removed. The LN draining the site of infection, however, regress slowly in cellularity and remain enlarged for several weeks. Likewise, non-draining LN slowly recover in size to revert to the size of an uninfected animal. Independent of antigen persistence or not, LN size and distribution of circulating lymphocytes according to LN size can explain why recovery takes time.

In our system, the host immune response to the infection did not compensate for the reduction of lymphocytes in superficial skin LN. As such, the expansion of mLN in the gut occur at the expense of peripheral LN cellularity, with a negative correlation between iLN and mLN sizes following *H*. *polygyrus* infection (Fig 7A). Thus, the muted immune response to BCG infection in skin is likely explained by the overall reduction in lymphocyte numbers in peripheral skin LN of worm-infected individuals. A model in which a redistribution of the circulating lymphocytes amongst LN occurs following *H*. *polygyrus* infection and is maintained in the chronic phase by the sizes of LN can be envisage (Fig 7B). We can assume that there is a cost benefit to limiting the size of the lymphocyte pool, saving the host’s energy as well as decreasing the risks of immune-associated pathologies. However, limiting lymphocyte numbers may compromise the ability of the host to mount multiple immune reactions. In chronic intestinal worm infection, the atrophy in superficial LN can result in a reduced capacity to initiate an immune response elsewhere resulting in a diminished immune response to a secondary infection in the periphery. This competition for cells provide an explanation to why host immunity to co-infections and vaccination can be dampened in animals, including humans, with chronic intestinal worms, which does not involve Th2 or regulatory cells.

The competition for lymphocytes in our model does not exclude that circulating cells also redistribute to Peyer’s patches (PP) or the small intestine (SI). Such involvement would not change our interpretation, but rather more broadly implicate the gut associated lymphoid tissue in the redistribution of lymphocytes during chronic infection with intestinal worms. However, the mLN are most likely the main receivers of redistributes cells, since the number of lymphocytes in PP and SI are fewer compared to the mLN [38], thus their contribution less according to our model. Further local immune dampening effects by the worms may also have a more direct effect in the SI preventing PP from expanding overly much [39].

De-worming may be a strategy to boost responsiveness to vaccines in areas where high worm burdens prevails. In support of this, worm-infected individuals treated with albendazol were found to have better immune responses to BCG and malaria parasites compared to those left untreated [7,25]. Our data show that de-worming can restore LN cellularity and responses to a BCG injection given in the skin. Importantly our data show that time is needed for the peripheral LN to recover cellularity. Indicating that time between de-worming and vaccination may be an important consideration for maximizing the outcome of the subsequent vaccination.

## Materials and methods

### Mice and lymph node weight

C57BL/6 congenic CD45.1 (Ly5.1), C57BL/6-GFP, P25-TCRTg RAG-1-/- [40] x RAG-1-/- ECFP or EGFP (originally provided by Dr. R. Germain, NIAID, USA) mice were bred and maintained under specific-pathogen-free conditions at MTC or KM-Wallenberg facilities, Karolinska Institutet (KI), Sweden. Wild type C57BL/6 mice were either bred at MTC/KI or purchased from Janvier Labs (Renne, France). Both female and male mice were used. Mice were age and sex matched.

### Infections

All infections were performed in wild type (C57BL/6 or congenic Ly5.1/CD45.1) mice. If not otherwise mentioned mice were infected by oral gavage with 200 *H*. *polygyrus* L3 larvae, obtained as described previously [41,42] at 4–5 weeks of age. The worm-infections were considered chronic after 28 days. At the end of each experiment, the worm burden was estimated by counting viable worms that had migrated out of the opened intestine through a fine net into a tube containing RPMI-1640 at 37°C within 3–4 hours.

*Mycobacterium bovis* Bacillus Calmette-Guérin (BCG) strain SSI 1331 (Statens Serum Institut, Denmark) was expanded in 7H9 medium and inoculated at 1×10^6^ colony forming units (CFU) in the footpad as described elsewhere [43].

### Estimation of LN size and cell counting

Superficial skin-draining (popliteal, pLN; inguinal, iLN; and axillary, aLN) and total mesenteric LN (mLN) were collected in PBS at various time points following *H*. *polygyrus* and BCG infections. Lymph node (LN) weight was determined using an analytical balance (Denver Instruments SI114). Cellularity was estimated in single cells suspension of LN, prepared by crushing the LN with a pestle or to determine stromal cells following tissue digestion, as described elsewhere [44]. Lymphocytes were counted using trypan blue exclusion either by microscopy using a haemocytometer, by an automated cell counter cell (Countess II, Life Technologies) or by FACS using beads (Countbright, Absolute bright count, Thermo Scientific). Since single cells suspension of LN are >95% lymphocytes (S4 Fig) and differences between counting methods was considered not to significantly impact the results and was not corrected for. For estimation of cells in blood, 20μl venous blood was collected from the tail into EDTA treated tubes at different times points following *H*. *polygyrus* infection. Cell counts were determined using an automated haematology analyser (Mindray BC-2800Vet).

### Flow cytometry (FACS)

Single-cell suspensions from tissues were incubated with 0.5 mg/ml anti-mouse FcγIII/II receptor (2.4G2) (BD Biosciences) for 10 min followed by various combinations of flourochrome-conjugated rat anti-mouse monoclonal antibodies (BD-Pharmingen) specific for CD45 (30-F11), MHCII I-A/I-E (M5/114.15.2), CD11b (M1/70), CD11c (HL3), CD3 (17A2), CD19 (eBio1D3), CD4 (RM4-5), CD8 (53–6.7), CD44 (IM7), CD69 (H1.2F3), CD62L (MEL-14), B220 (RA3-6B2), podoplanin (8.1.1), CD31 (MEC13.3) for 35 min in FACS buffer (2% FCS in 5mM EDTA, 0.1% azide). For analysis FoxP3 (FJK-16s) and Ki67 (SolA15) (eBioscience) FoxP3 and Ki67 staining set were used according to manufacturer’s instructions (eBioscience). To detect cytokine production, cells were restimulated with PMA/Ionomycin (Sigma) and treated with Brefeldin A (Sigma), prior to intracellular IFNγ (XMG1.2, BD Pharmingen) and surface staining, as previously described [43]. Irrelevant isotype-matched antibodies were used to determine levels of non-specific binding. The sample were acquired on LSRII (BD Bioscience) and analysed by FlowJo version 10 (Treestar), using the gating strategies shown in S4 Fig.

### Cell transfers and treatments

For transfer of cells, superficial LNs from young adult (<8 weeks) mice were collected and total lymphocytes harvested by making single cell LN suspension as described above. To enable tracking of lymphocytes we either used congenic cell, GFP-expressing cell or cells labelled with CFSE as previously described [45]. FACS determined the composition of transferred cell. The number of cells and time of transfer are indicated in respective figures.

For deworming, mice were treated with pyrantel pamoate per oral gavage 40mg/mice, 3 times with one-day interval. Efficacy of de-worming was checked by assessment of egg in faeces samples. De-worming was considered effective when no eggs were detected.

Fingolimod treatment: 3 mg/kg FTY720 (Sigma Aldrich) were injected *i*.*p*. daily for total 28 days starting one day before infection with *H*. *polygyrus*.

IL-7 treatment: Mice were injected *i*.*p*. with 4μg/mouse recombinant murine IL-7 (R&D Systems, 407-ML/CF) / every second day for 28 days, starting 4 hours after infection with *H*. *polygyrus*.

### IL-7 ELISA

Serum was separated from venous blood collected from mice with or without *H*. *polygyrus* infection was collected and stored at -80°C until assessment. Serum concentrations were determined using the IL-7 ELISA kit (M7000, R&D System) following manufacturers’ instructions.

### Quantification PCR and RNA sequencing

Total RNA was extracted from inguinal LN using Trizol (Thermo Scientific) according to manufacturer’s instructions and the RNA concentration was determined by Nanodrop spectrometry (NanoDrop 2000c, Thermo Scientific).

For RNA sequencing the RNA quality was determined using a bio-analyzer (CliperLabchip GX) and three samples from each group with RIN values above 6.0 were selected for further processing. 2 μg of RNA was used to construct sequence library using Illumina TruSeq Stranded mRNA library Prep kit (Illumina). Sequencing was then performed on a HiSeq 2500 (Hiseq Control Software2.2.58/RTA 1.18.64) with a 2x 125 bp paired-end reads. Reads were mapped to the Mouse genome assembly, build NCBIM37, with Tophat/2.0.4, merged and duplicates were removed using picard-tools/1.29 in samtools. 5.3–8.3 million mappable reads were obtained from the samples. Gene constructs were generated using htseq/0.6.1 on bam files with duplicates included. Fragment Per Kilobase of transcript per Million mapped reads (FPKM) for genes and transcripts were generated using cufflinks/2.1.1. Correlations within replicate groups were used to assess the background FPKM, which was set to 3. A transcript was thereafter only considered if it passed the following criteria A) evidence of the transcript (FPKM > 3) in at least three of six animals, B) not being of non-coding nature (filtered using the available ncRNA information available in the ENSEMBL GRCm38.p5 genome assembly). In the instance where a transcript displayed FPKM <3 in three animals or less, those values were adjusted to 3. FPKM values were thereafter Log_2_ transformed and used in the make.heatmap1 function distributed in the R-project NeatMap package to generate non-clustered heatmaps with median row normalizations to visualize relative gene expression. Relative expression of selected genes is shown based on assumed association with inflammation, lymphocytes, cell migration, cell death/survival and extra cellular matrix, all deemed to be of possible relevance to outcome of skin BCG infection. Gene expression data is available at NCBI, SRA (BioProject accession PRJNA433170). For conventional RT-qPCR first strand cDNA generation was performed using Invitrogen Superscript. Real-time PCR was performed on CFX384 (Bio-Rad) using FAM-MGB labelled primer/probe for mRNA of interest (all best coverage TaqMan gene expression assay, Applied Biosystems) or SYBR green (Sigma) identification of double stranded DNA following amplification using cDNA specific primers (S1 Table).

Expression of HRPT was used as housekeeping (hk) reference and the relative expression of gene expression was calculated using the 2^-ΔΔCt^ method.

### Immunofluorescence microscopy

Popliteal lymph nodes were placed in OCT compound, frozen on dry ice, and stored in -20°C until sectioning. 8 μm thick sections were blocked for 1 hour in 2.5% normal goat serum (Jackson ImmunoResearch) followed by incubation with directly conjugated (rat anti-CD3 AF647 clone 17A2 and rat anti-B220 AF488 clone RA3-6B2; Biolegend) and unconjugated polyclonal rabbit anti-collagen IV (Abcam) antibodies in goat serum over night at 4°C. Sections were then incubated with secondary antibody (chicken anti-rabbit AF594; Invitrogen) for 1 hour in goat serum and mounted in mounting media with DAPI (Vectachield, Vector Laboratories). Images were acquired with a confocal microscope (Zeiss LMS 800) and analysed using Fiji (ImageJ) software.

### Statistical analysis

Statistical analyses were done using PRISM versions 6 and 7 (GraphPad). Groups consisted of five or more mice at the start of an experiment. Samples/animals were excluded from the analysis if the worm infection was considered a failure (i.e. worm burden in an untreated mouse at termination log^10^ less than normal, which typically equals≤10 worms per gut). In groups that received treatments, outliers were removed following Grubb’s test to define single outliers (GraphPad online tools). All exclusion criteria were set before the start of the study. For comparison between two groups, we used student’s *t*-test, or non-parametric Mann-Whitney U test if samples diverged from normal distribution. Kruskall-Wallis test was used for multiple comparisons. Correlations were done using Pearson’s linear correlation test. Mean and SD are shown if not otherwise indicated. Statistically significant differences between groups are indicated as *p<0.05, **p<0.01, ***p<0.001.

### Ethics statement

Animal experiments were conducted in accordance to national regulations outlined in L150 (Föreskrifter och allmänna råd om Försöksdjur, SJVFS 2012:26). Animals were euthanized by cervical dislocation in accordance with approved ethical protocol. Isofluran anesthesia was used for footpad injections. Ethical approval was granted by the regional ethical board (Stockholms djurförsöksetiska nämnd) permit number N171/14 with amendment N131/16 and exemption from L150 Dnr 5.2.18-7344/14 approved by the Swedish Board of Agriculture.

## Supporting information

S1 Table List of primers used. (DOCX)

S1 Fig Inguinal LN (iLN) cellularity in uninfected and *H*. *polygyrus* infected C57BL/6 mice 28 and 90 days after infection **(A)**. Effect of different infectious doses on iLN cellularity 28 days after infection in Ly5.1 mice **(B)**. Data from two pooled experiments are shown. Body weight of C57BL/6 mice when infected at 4–5 weeks of age and 28 days after infection **(C)**. Average daily food intake in C57BL/6 mice during the course of 28 days *H*. *polygyrus* infection **(D)**. LN cellularity 28 days after infection when given to 4-week old adolescent (4W) and 9-week old adult (9W) C57BL/6 mice at the time of infection **(E)**. Effector and memory CD4 T cells **(F, G)** and CD8 T cells **(H, I)** in iLN four weeks after *H*. *polygyrus* infection. Frequencies of naïve, effector and memory CD4 **(J-L)** and CD8 T cells **(M-O)** in iLN. Cell were gated on viable CD3+ singlet cells, data shown is from one of two or more experiments performed with similar results.
(TIF)

S2 Fig Cell counts in suspensions of spleens from *H*. *polygyrus* and uninfected C56BL/6 mice 28 days after infection. Red cells were removed using ACK lysis buffer before cells were counted **(A)**. Frequency of lymphocyte subsets in the transferred (GFP+) cell population in inguinal LN, iLN **(B-F)** and mesenteric, mLN **(G-K)** 4 hours after *i*.*v*. cell transfer into recipient C57BL/6 mice. For A-K 1x10^6^ cells obtained from peripheral LN of C57BL/6-GFP were injected 4 hours before mice were euthanized and analysis performed. Number (**L, M**) and frequencies (**N, O**) of transferred (CFSE+) cell in mLN (**L, N**) and popliteal, pLN (**M, O**), 12 hours after cell transfer. For L-O, the total LN cells from C57BL/6 mice were stained with CFSE and 1x10^6^ CSFE labelled cells injected *i*.*v*. 12 hours before mice were euthanized and analysis performed.
(TIF)

S3 Fig Recovery of lymphocytes in skin draining LN. Effect of rIL-7 treatment on cell subset in iLN from mice infected with *H*. *polygyrus* or worm free **(A-H)**. Effect of de-worming on cell subsets in iLN from mice infected with *H*. *polygyrus* or worm free, 10 days after de-worming **(I-P)** and 21 days after de-worming **(Q-X)**. Total CD3+ cell **(A, I, Q)**; total CD19+ cells **(B, J, R)**; total CD4+ T cells **(C, K, S)**; total CD8+ T cells **(D, L, T)**; CD62L^low^CD44^hi^ CD4 T cells **(E, M, U)**; CD62L^hi^CD44^int/low^ CD4 T cells **(F, N, V)**; CD62L^low^CD44^hi^ CD8 T cells **(G, O, W)**; CD62L^hi^CD44^low^ CD8 T cells **(H, P, X)**.
(TIF)

S4 Fig Gating strategy used in FACS analysis of cell subsets. (**A**): Cell were first gated by side (SSC) and forward (FSC) scatter profiles to exclude debris. Then doubles were excluded and only singlets (>99%) used for further analysis. Dead cells (10–30%) were removed using Live/Dead stain (Thermo Fischer). Live cells were 96–99% CD45+ cells. Leukocytes (CD45+) were analysed further for expression of B cell (CD19) and T cell (CD3) markers. T cell were divided into CD4 and CD8 and CD103+, CD62L+ and CD44+ cells were measured in gated CD4+ or CD8+ T cells as shown in the lower panels. (**B**): P25-TCRTg cells detected by expression of eGPF in cells gated as CD45+, as shown in figure A. Intracellular detection of IFNγ **(C)** and Ki67 (**D**) in CD4 T cells gated as shown in figure A.
(TIF)
