# Supplementary material for: Atrophy of skin-draining lymph nodes predisposes for impaired immune responses to secondary infection in mice with chronic intestinal nematode infection
Source: PLoS Pathog. 2018 May 17;14(5):e1007008. doi: 10.1371/journal.ppat.1007008 (PMC5957330; doi:10.1371/journal.ppat.1007008)
Supplement: S1 Table — (DOCX) [file ppat.1007008.s001.docx]

**S1 Table: List of primers used**

| Gene name | Forward 5’ | Reverse 3’ | Reference |
| --- | --- | --- | --- |
| *hprt* | CTTCCTCCTCAGACCGCTTT | ACCTGGTTCATCATCGCTAA | [1] |
| *gata3* | TTATCACGCCCACGCGAAGG | CATTCGCGTTCCTCCTCCAGA | [2] |
| *il-4* | AACGAGGTCACAGGAGAAGG | TCTGCAGCTCCATGAGAACA | [3] |
| *il-7* | CGATGAATTGGACAAAATGACAGG | AACTTGCGAGCAGCACGATTTAG | [4] |
| *il-10* | ACTGCTATGCTGCCTGCTCTT | CCGCAGCTCTAGGAGCATGT | [5] |
| *tgfβ* | TTGCTTCAGCTCCACAGAGA | TGGTTGTAGAGGGCAAGGAC | [6] |
| *glycam-1* | AGCTGGAAGATCGAAAGTCCG | AGCTGGAAGATCGAAAGTCCG | [7] |
| *icam-1* | CAATTTCTCATGCCGCACAG | AGCTGGAAGATCGAAAGTCCG | [8] |
| *ccl19* | ATGTGAATCACTCTGGCCCAGGAA | AAGCGGCTTTATTGGAAGCTCTGC | [9] |
| *ccl21* | GAGCTATGTGCAAACCCTGAGGA | TGAGGGCTGTGTCTGTTCAGTTCT | [9] |
| *foxp3* | *TaqMan Primer-Probe (no 4331182, Life Technologies)* | | |

**S1 TABLE REFERENCES**

1. Weinreich MA, Takada K, Skon C, Reiner SL, Jameson SC, Hogquist KA. KLF2 transcription-factor deficiency in T cells results in unrestrained cytokine production and upregulation of bystander chemokine receptors. Immunity. 2009;31(1):122-30. doi: 10.1016/j.immuni.2009.05.011. PubMed PMID: 19592277

2. Matsuo H, Kondo Y, Kawasaki T, Tokuyama S, Imamura N. Borrelidin Isolated from Streptomyces sp Inhibited Adipocyte Differentiation in 3T3-L1 Cells via Several Factors Including GATA-Binding Protein 3. Biol Pharm Bull. 2015;38(10):1504-11. PubMed PMID: WOS:000362047200009.

3. Choi J, Choi BK, Kim JS, Lee JW, Park HA, Ryu HW, et al. Picroside II Attenuates Airway Inflammation by Downregulating the Transcription Factor GATA3 and Th2-Related Cytokines in a Mouse Model of HDM-Induced Allergic Asthma (vol 11, e0167098, 2016). Plos One. 2017;12(1). doi: ARTN e0170832

10.1371/journal.pone.0170832. PubMed PMID: WOS:000392405300180.

4. Hess E, Duheron V, Decossas M, Lezot F, Berdal A, Chea S, et al. RANKL induces organized lymph node growth by stromal cell proliferation. J Immunol. 2012;188(3):1245-54. doi: 10.4049/jimmunol.1101513. PubMed PMID: 22210913.

5. Yang L, Han Z, Tian L, Mai P, Zhang Y, Wang L, et al. Sphingosine 1-Phosphate Receptor 2 and 3 Mediate Bone Marrow-Derived Monocyte/Macrophage Motility in Cholestatic Liver Injury in Mice. Sci Rep. 2015;5:13423. doi: 10.1038/srep13423. PubMed PMID: 26324256; PubMed Central PMCID: PMCPMC4555045.

6. Li J, Geng S, Xie X, Liu H, Zheng G, Sun X, et al. Caveolin-1-mediated negative signaling plays a critical role in the induction of regulatory dendritic cells by DNA and protein coimmunization. J Immunol. 2012;189(6):2852-9. doi: 10.4049/jimmunol.1102828. PubMed PMID: 22904311.

7. Hou ZY, Bailey JP, Vomachka AJ, Matsuda M, Lockefeer JA, Horseman ND. Glycosylation-dependent cell adhesion molecule 1 (GlyCAM 1) is induced by prolactin and suppressed by progesterone in mammary epithelium. Endocrinology. 2000;141(11):4278-83. doi: DOI 10.1210/en.141.11.4278. PubMed PMID: WOS:000089970300044.

8. Ren G, Zhao X, Zhang L, Zhang J, L'Huillier A, Ling W, et al. Inflammatory cytokine-induced intercellular adhesion molecule-1 and vascular cell adhesion molecule-1 in mesenchymal stem cells are critical for immunosuppression. J Immunol. 2010;184(5):2321-8. doi: 10.4049/jimmunol.0902023. PubMed PMID: 20130212; PubMed Central PMCID: PMCPMC2881946.

9. Dende C, Meena J, Nagarajan P, Panda AK, Rangarajan PN, Padmanaban G. Simultaneously targeting inflammatory response and parasite sequestration in brain to treat Experimental Cerebral Malaria. Sci Rep. 2015;5:12671. doi: 10. 1038/srep12671. PubMed PMID: 26227888; PubMed Central PMCID: PMCPMC4521148.
