# Supplementary figures and images for: Atrophy of skin-draining lymph nodes predisposes for impaired immune responses to secondary infection in mice with chronic intestinal nematode infection
Source: PLoS Pathog. 2018 May 17;14(5):e1007008. doi: 10.1371/journal.ppat.1007008 (PMC5957330; doi:10.1371/journal.ppat.1007008)

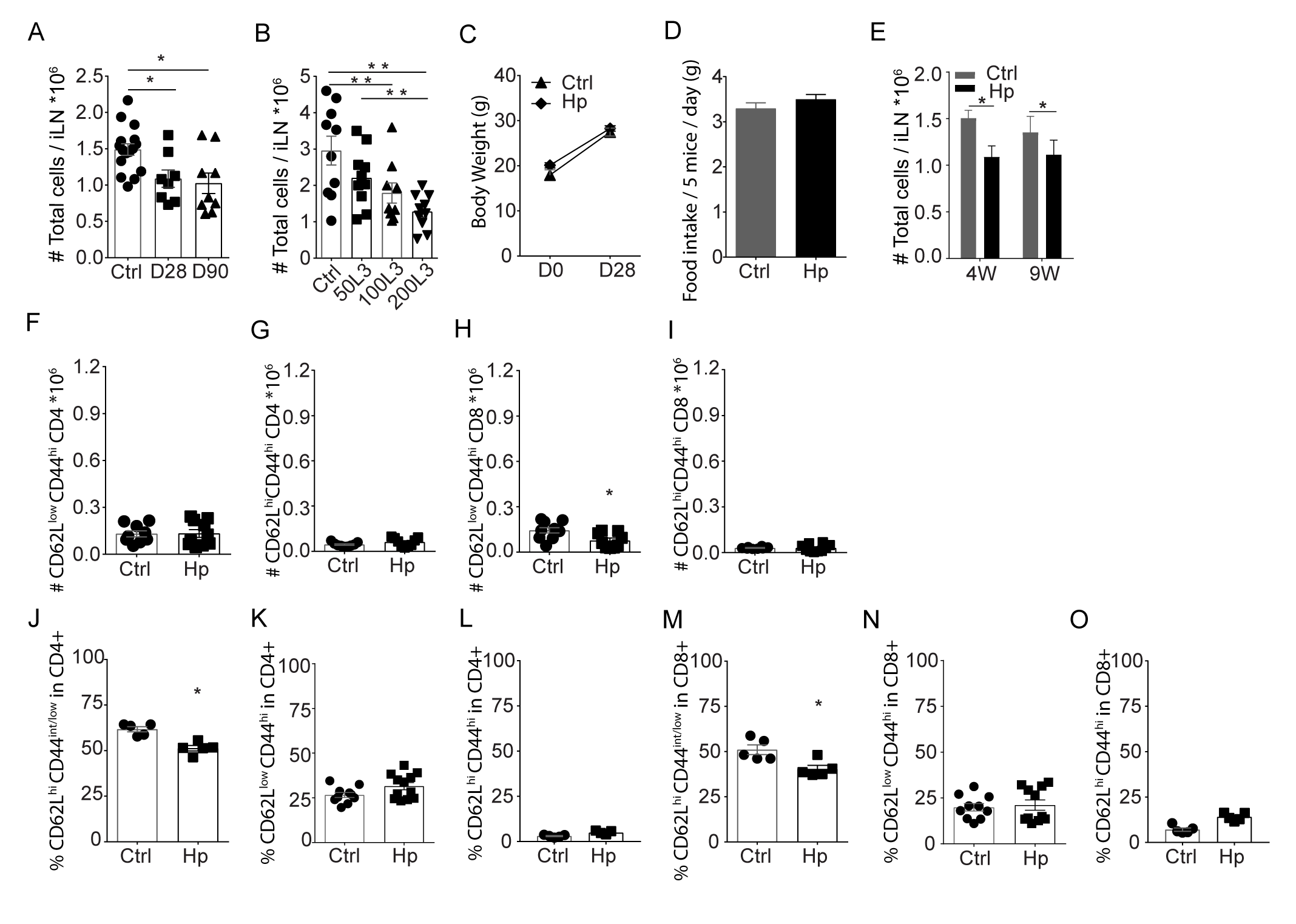

Supplement: S1 Fig — Inguinal LN (iLN) cellularity in uninfected and H. polygyrus infected C57BL/6 mice 28 and 90 days after infection (A). Effect of different infectious doses on iLN cellularity 28 days after infection in Ly5.1 mice (B). Data from two pooled experiments are shown. Body weight of C57BL/6 mice when infected at 4–5 weeks of age and 28 days after infection (C). Average daily food intake in C57BL/6 mice during the course of 28 days H. polygyrus infection (D). LN cellularity 28 days after infection when given to 4-week old adolescent (4W) and 9-week old adult (9W) C57BL/6 mice at the time of infection (E). Effector and memory CD4 T cells (F, G) and CD8 T cells (H, I) in iLN four weeks after H. polygyrus infection. Frequencies of naïve, effector and memory CD4 (J-L) and CD8 T cells (M-O) in iLN. Cell were gated on viable CD3+ singlet cells, data shown is from one of two or more experiments performed with similar results. (TIF) [file ppat.1007008.s002.tif]

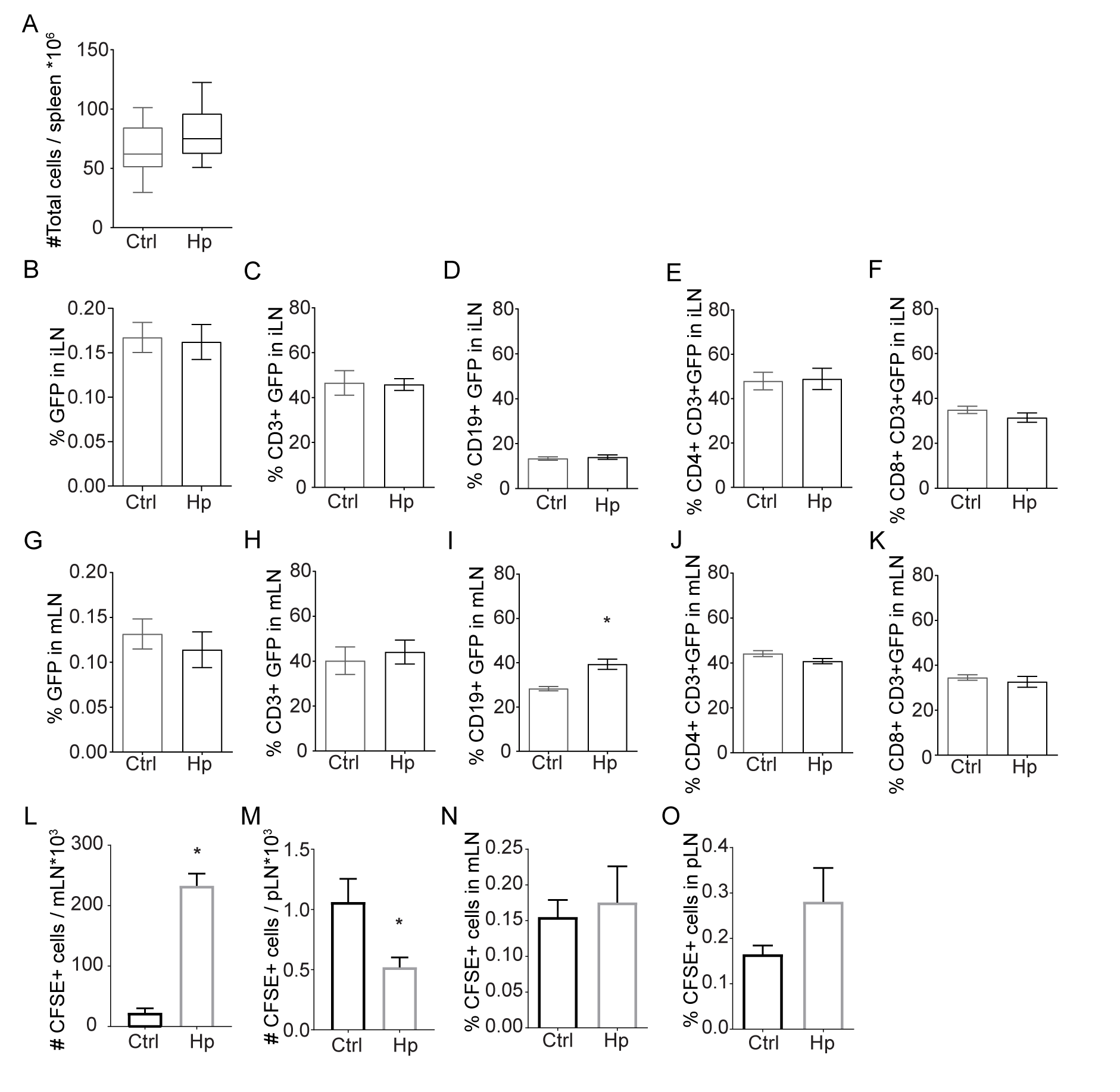

Supplement: S2 Fig — Red cells were removed using ACK lysis buffer before cells were counted (A). Frequency of lymphocyte subsets in the transferred (GFP+) cell population in inguinal LN, iLN (B-F) and mesenteric, mLN (G-K) 4 hours after i.v. cell transfer into recipient C57BL/6 mice. For A-K 1x10^6^ cells obtained from peripheral LN of C57BL/6-GFP were injected 4 hours before mice were euthanized and analysis performed. Number (L, M) and frequencies (N, O) of transferred (CFSE+) cell in mLN (L, N) and popliteal, pLN (M, O), 12 hours after cell transfer. For L-O, the total LN cells from C57BL/6 mice were stained with CFSE and 1x10^6^ CSFE labelled cells injected i.v. 12 hours before mice were euthanized and analysis performed. (TIF) [file ppat.1007008.s003.tif]

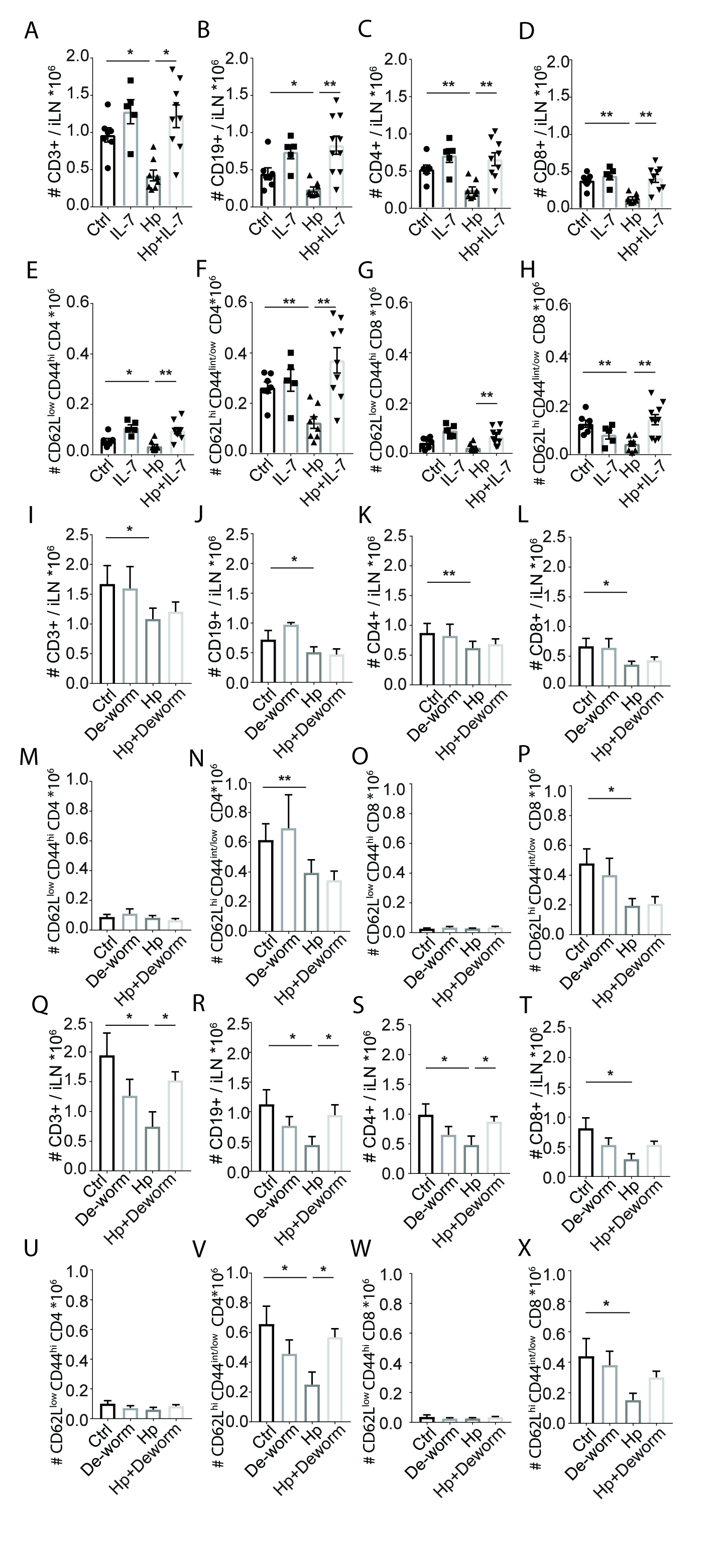

Supplement: S3 Fig — Effect of rIL-7 treatment on cell subset in iLN from mice infected with H. polygyrus or worm free (A-H). Effect of de-worming on cell subsets in iLN from mice infected with H. polygyrus or worm free, 10 days after de-worming (I-P) and 21 days after de-worming (Q-X). Total CD3+ cell (A, I, Q); total CD19+ cells (B, J, R); total CD4+ T cells (C, K, S); total CD8+ T cells (D, L, T); CD62LlowCD44hi CD4 T cells (E, M, U); CD62LhiCD44int/low CD4 T cells (F, N, V); CD62LlowCD44hi CD8 T cells (G, O, W); CD62LhiCD44low CD8 T cells (H, P, X). (TIF) [file ppat.1007008.s004.tif]

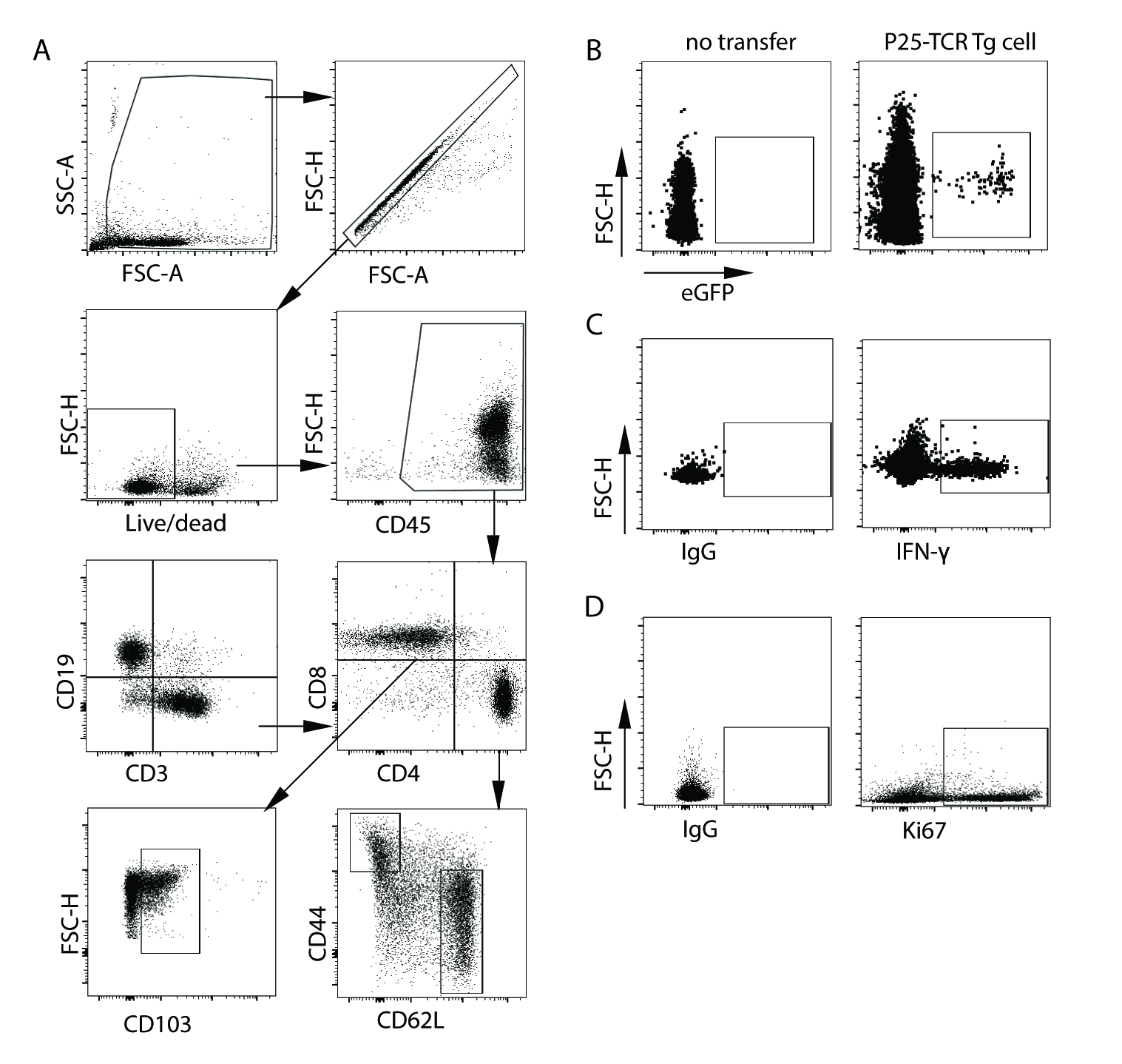

Supplement: S4 Fig — (A): Cell were first gated by side (SSC) and forward (FSC) scatter profiles to exclude debris. Then doubles were excluded and only singlets (>99%) used for further analysis. Dead cells (10–30%) were removed using Live/Dead stain (Thermo Fischer). Live cells were 96–99% CD45+ cells. Leukocytes (CD45+) were analysed further for expression of B cell (CD19) and T cell (CD3) markers. T cell were divided into CD4 and CD8 and CD103+, CD62L+ and CD44+ cells were measured in gated CD4+ or CD8+ T cells as shown in the lower panels. (B): P25-TCRTg cells detected by expression of eGPF in cells gated as CD45+, as shown in figure A. Intracellular detection of IFNγ (C) and Ki67 (D) in CD4 T cells gated as shown in figure A. (TIF) [file ppat.1007008.s005.tif]
